# Transcriptome Analysis Reveals the Molecular Mechanisms Underlying Growth Superiority in a Novel *Gymnocypris* Hybrid, *Gymnocypris przewalskii* ♀ × *Gymnocypris eckloni* ♂

**DOI:** 10.3390/genes15020182

**Published:** 2024-01-29

**Authors:** Yun Zhao, Junming Zhou, Yanzhen Dong, Dayong Xu, Dongming Qi

**Affiliations:** Key Laboratory of Plateau Wetland Ecology and Environmental Protection, Xichang University, Xichang 615013, China; 1901130065@nbu.edu.cn (Y.Z.); xcc04000019@xcc.edu.cn (Y.D.); xcc04000017@xcc.edu.cn (D.X.); xcc04000029@xcc.edu.cn (D.Q.)

**Keywords:** hybrid F1, growth performance, transcriptome, *Gymnocypris eckloni*, *Gymnocypris przewalskii*

## Abstract

Artificial hybrid breeding can optimize parental traits to cultivate excellent hybrids with enhanced economic value. In this study, we investigated the growth performance and transcriptomes of *Gymnocypris przewalskii* (♀) and *Gymnocypris eckloni* (♂) and their F1 hybrid fishes. Hatched individuals of *G. przewalskii* (GP) and *G. eckloni* (GE) of the same size and their F1 hybrids (GH) were separately cultured for eight months in three cement tanks (*n* = 3). The growth indexes were measured, which showed that the growth rate of the groups was GE > GH > GP, while the survival rate was GH > GE > GP. The RNA-Seq data analysis of the muscles from the three *Gymnocypris* fish strains revealed that gene transcription has a significant impact on F1 hybrid fish and its parents. The differentially expressed genes (DEGs) in GH show less differences with GP, but more with GE. qRT-PCR was used to confirm the expression profiles of the chosen DEGs, and the results showed positive correlations with the RNA-seq data. KEGG enrichment results indicated that the DEGs were related to a variety of molecular functions, such as glycolysis/gluconeogenesis, arachidonic acid formation, citrate cycle, and the MAPK, PI3K-Akt, or mTOR signal pathways. Subsequent analysis indicated that there may be a significant correlation between the differential expression of IGF2 and a difference in the growth of GE and GP.

## 1. Introduction

The rapid advancement of next-generation sequencing technologies has promoted the use of transcriptomics in the aquaculture sector [1,2]. The transcriptome provides a blueprint of the genes that are actively being transcribed in an organism, thus uncovering the molecular mechanisms that potentially govern specific biological processes or drive metabolic and genetic changes in it [3]. Thus, the transcriptome is becoming a powerful tool to reveal the growth advantage of hybrid generations in many aquatic organisms of economic relevance [4,5].

Crossbred animals often feature heterosis, which is the increased vitality and performance of the progeny compared to the parents [6]. Interspecific hybridization is a useful strategy to achieve heterosis and improve the phenotypes and the genotypes of the offspring. Due to the combination of beneficial traits from both male and female parents, the hybrids exhibit heterosis in growth rate, survival, and disease resistance [7]. Current hybridization experiments on fish have mostly been conducted between different genera, subfamilies, families, or orders [6,8,9,10]. Liu [11] obtained progenies of diploid hybrids with rapid growth rates that were derived from female red crucian carp and male common carp. Sun’s [4] research results demonstrated that a novel hybrid offspring, the Hulong grouper, which was a cross between *Epinephelus fuscogutatus* (♀) and *Epinephelus lanceolatus* (♂), exhibited significant growth superiority over its female parent. Yang [12] crossed two species of *Acipenser baeri* ♀ and *Acipenser schrenckii* ♂, and their hybrid F1 had a growth advantage. Wang [6] generated live hybrids from crosses between *Megalobrama amblycephala* (order Cypriniformes) and *Siniperca chuatsiv* (order Perciformes) species, which presented an obvious growth advantage over the female parent.

*G. przewalskii*, belonging to the Schizothoracinae subfamily and genus *Gymnocypris*, is a unique indigenous fish and aquatic biological resource in the Qinghai Tibet Plateau (elevation 3200 m) with ecological and economic value [13,14,15]. Due to its unique geographical environment, *G. przewalskii* has excellent characteristics of resistance to salt–alkaline conditions and cold temperatures, as well as low oxygen tolerance, but its growth performance is very slow [16]. *Gymnocypris eckloni* belongs to the subfamily Schizothoracinae; it is a relative of *G. przewalskii* [17], it is mainly distributed in the Yellow River system, [18] and it grows faster than *G. przewalskii*. In recent years, due to anthropogenic influences, such as the drainage of wild germplasm resources, the loss of the wild germplasm of *G. przewalskii* has become a serious issue. Experiments based on artificially breeding hybrid species as part of genetic diversity research have helped optimize parental traits to cultivate excellent hybrids with enhanced economic value. This also helps achieve the effective utilization and protection of germplasm resources of the two *Gymnocypris* species.

This work is the first to document heterosis between growth and survival rates in F1 hybrid fish. Additionally, we compared the transcriptomes of F1 hybrid *Gymnocypris* and their parents to explore the molecular mechanism of the different performances in the growth of F1 hybrids. Our investigation also offers theoretical justification for the breeding of hybrid *Gymnocypris*.

## 2. Material and Method

### 2.1. Fish and Sample Preparation

*G. przewalskii* (♀, GP), *G. eckloni* (♂, GE), and their F1 hybrid (*Gymnocypris*, GH) were cultivated under the same breeding conditions in XiDe ZhengYuan Fish Farm in Liangshan Prefecture, Sichuan Province, China. After hatching, 500 of each *Gymnocypris* species were cultured separately in net cages in filtered water. The same conditions of freshwater, temperature (13 °C), food (expanded formula feed was showed in Table S1), and light (on at 6:00 and off at 18:00) were maintained for 6 months. At the beginning and end of breeding, 15 fish were randomly taken for growth index measurement. Subsequently, three individuals from GH (length, 8.65 ± 0.94 cm), GE (length, 12.16 ± 1.37 cm), GP (length, 7.42 ± 0.24 cm) were randomly selected for further experiments. Fresh muscle tissues were collected and immediately stored in liquid nitrogen. All experiments involving animals were conducted according to the guidelines and approval of the respective Animal Research and Ethics Committees of Xi Chang University.

### 2.2. RNA Extraction, Library Construction, and High-Throughput Sequencing

Total RNA was extracted from the muscle tissues with the Trizol reagent (Takara Bio, Otsu, Japan). RNA purity and quantity were evaluated using a NanoDrop 2000 spectrophotometer (Thermo Scientific, from Waltham, MA, USA), and the total RNA was measured using ≥1 μg, OD260/280 in 1.8–2.2, Agilent2100 RIN ≥ 7. Its integrity was assessed using the Agilent 2100 Bioanalyzer (Agilent Technologies, Santa Clara, CA, USA). RNA libraries were then constructed using VAHTS Universal V6 RNA-seq Library Prep Kit for Illumina according to the manufacturer’s instructions. The libraries were finally sequenced using the Illumina NovaSeq 6000 platform, and 150 bp-long paired-end reads were generated. Transcriptome sequencing and analysis were conducted by OE Biotech Co., Ltd. (Shanghai, China).

### 2.3. RNA Sequencing Analysis

Raw data on RNA were gathered in the FASTQ format, which was then processed using fastp (Version 20.1, length required 50). The reads containing poly-N and the low-quality reads were removed to obtain clean reads. After removing the adaptor and low-quality sequences, clean reads were assembled into expressed sequence tag clusters (called contigs). They were subjected to de novo assembly into transcripts in Trinity (Version 2.4, seqType fq SS_lib_type RF) via the paired-end method. The longest transcript was chosen as a unigene based on the similarity and length of the subsequent analyses. The function of the unigenes was annotated using the Swiss–Prot database with the diamond tool and a threshold of e < 1 × 10^−5^. The proteins with the highest hits to the unigenes were used to assign functional annotations. In addition, the unigenes were mapped against the Kyoto Encyclopedia of Genes and Genomes (KEGG) database to annotate their potential involvement in different metabolic pathways. After annotation, bowtie2 software (Version 2.3.3.1, reorder k30 t) was used to obtain the number of reads aligned to a unigene in each sample, and the eXpress software (Version 1.5.1, rf-stranded) was used to calculate the expression level of each unigene (FPKM). Differentially expressed unigenes (DEGs) between different groups were identified using the DESeq2 software (Version 1.20, *p* value < 0.05, |log2FoldChange| > 1), which calculated the difference and used NB (negative binomial distribution test) to evaluate the significance of the difference. Finally, the default filter conditions for DEG were set at *p* < 0.05 and foldChange > 2 or foldChange < 0.5. Hierarchical cluster analysis of DEGs was performed using R (version 3.2.0) to demonstrate the expression pattern of unigenes in the different experimental groups and samples. The KEGG pathway enrichment analysis of DEGs was also performed in R based on the hypergeometric distribution. Lastly, we used the oebiotech platform (https://cloud.oebiotech.cn/task/category/pipeline/, accessed on 25 September 2023) to draw the column and bubble diagrams of the significant enrichment terms.

### 2.4. Quantitative Real-Time PCR (qRT-PCR) Analysis

Trizol reagent was used to extract total RNA from the muscle samples (same as the transcriptome sequencing samples) following the manufacturer’s instructions. After RNA quality was detected by 1.5% agarose gel electrophoresis, a PrimeScript^®^RT Reagent Kit With gDNA Eraser (Takara Bio Inc., Shiga, Japan) was used to synthesize first-strand cDNA following the manufacturer’s instructions. The cDNA was used as a template for RT-PCR. Table S2 lists the primer used in this study. SYBR^®^-Premix Ex Taq™ was used to conduct qRT-PCR amplifications with 20 µL of reaction mixture on an ABI7500 Real-Time PCR Detection System (Applied Biosystems, from Foster City, CA, USA). All the experiments were conducted in triplicates. The comparative threshold cycle method (2^−ΔΔCT^) was used to analyze the expression levels of target genes with EF1α as the reference gene [19]. Each experiment involved three biological replicates.

### 2.5. Statistical Analyses

Data on the body length, weight, and survival rates of the juvenile fish were collected. These data were then used to calculate the following parameters:

Survival rate (%) = final number of animals/initial number of animals

Weight change rate (WR%) = (terminal weight − initial weight)/initial weight

Microsoft Excel 2010 (Microsoft, Washington, DC, USA) and SPSS version 22.0 (SPSS, Chicago, IL, USA) were used for statistical analyses. Comparisons between the three groups were performed using Student’s *t*-test, while one-way analysis of variance (ANOVA) and Duncan’s method were used for comparisons of multiple mean values between the experimental groups. Before statistical analyses, data were tested for normality (Shapiro–Wilk) and homoscedasticity (Bartlett’s test) of variances. *p* < 0.05 indicated a significant difference, and data were expressed as mean ± standard deviation (mean ± SD).

## 3. Results

Significant differences were observed in the terminal length and weight, survival rate, and weight increase rate (*p* < 0.05; Table 1). The indexes of terminal length and weight and weight increase rate showed that GE > GH > GP, while in survival rate, the order changed to GH > GE > GP.

A total of nine samples were used in this analysis. The Q30 of raw data for each sample was distributed from 93.40 to 94.35%. The effective data was distributed from 6.75 to 7.39 G, and the average GC content was 48.57 to 49.13% (Table S3).

**Figure 1:**
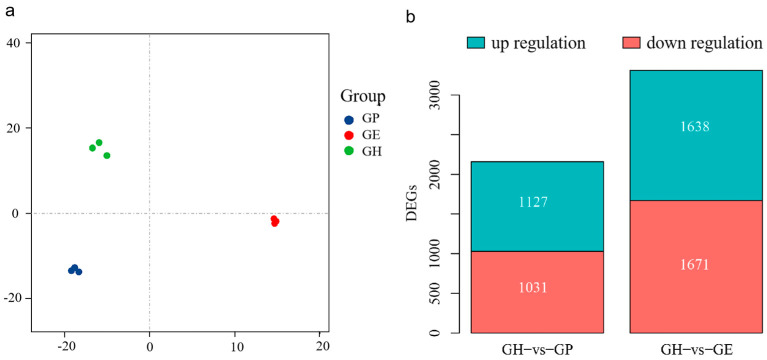
(**a**) The principal component analysis (PCA) plot shows the distribution of samples in different groups samples. (**b**) Bar graph shows the DEGs in GH vs. GP and GH vs. GE.

The principal component analysis (PCA) plot similarly showed that similar samples were grouped together (Figure 1a). DEGs between the F1 hybrid fish and its parents were screened out by filtering based on *p* < 0.05 and absolute log2 (ratio) ≥ 1 (Figure 1b). Results suggest that the number of DEGs in GH vs. GE was greater than in GH vs. GP. In GH vs. GP, 1031 genes were up-regulated and 1127 genes were down-regulated. In GH vs. GE, 1671 genes were up-regulated and 1638 genes were down-regulated (Figure 1b).

**Figure 2:**
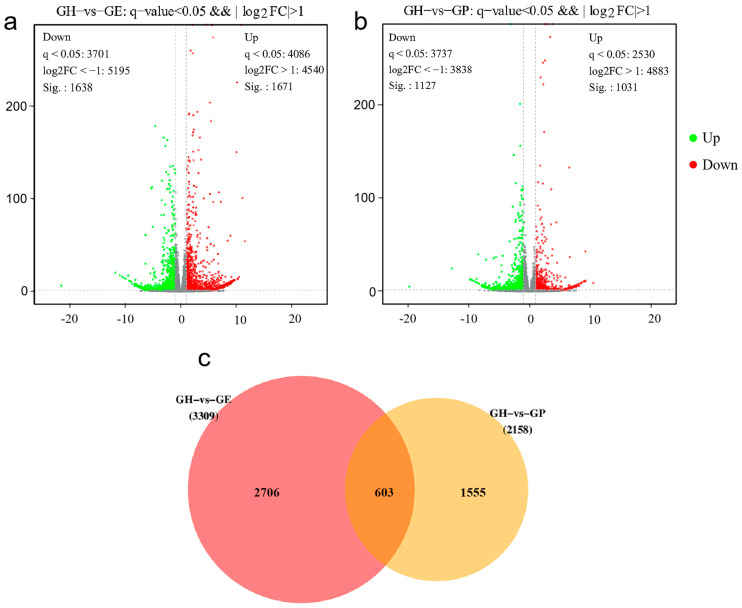
Volcano plots and a Venn diagram depicting differentially expressed genes (DEGs) in the hybrid F1 and its parents: (**a**) DEGs in GH vs. GE, (**b**) DEGs in GH vs. GP, and (**c**) Venn diagram illustrating up- and down-regulated genes in GH vs. GP and GH vs. GE. The grey dot of (**a**,**b**) means the expressed genes has no significant difference.

Venn analysis showed that the genes in the F1 hybrid group showed fewer differences with *G. przewalskii* and more differences with *G. eckloni*. In the GH vs. GE group, out of 2158 genes (*p* > 0.05), 454 were up-regulated (*p* < 0.05), and 250 genes were down-regulated (*p* < 0.05) (Figure 2a). In the GH vs. GP group, out of 3309 genes (*p* < 0.05), 967 were up-regulated (*p* < 0.05), and 1084 genes were down-regulated (*p* < 0.05) (Figure 2b). We also determined whether the DEGs between the parents overlapped with the genes that differ between each parent and the hybrid. We found 2158 and 3309 differentially expressed genes (DEGs) between GH vs. GP and GH vs. GE. The number of DEGs that overlapped between GH vs. GP and GH vs. GE was 603 (Figure 2c).

**Figure 3:**
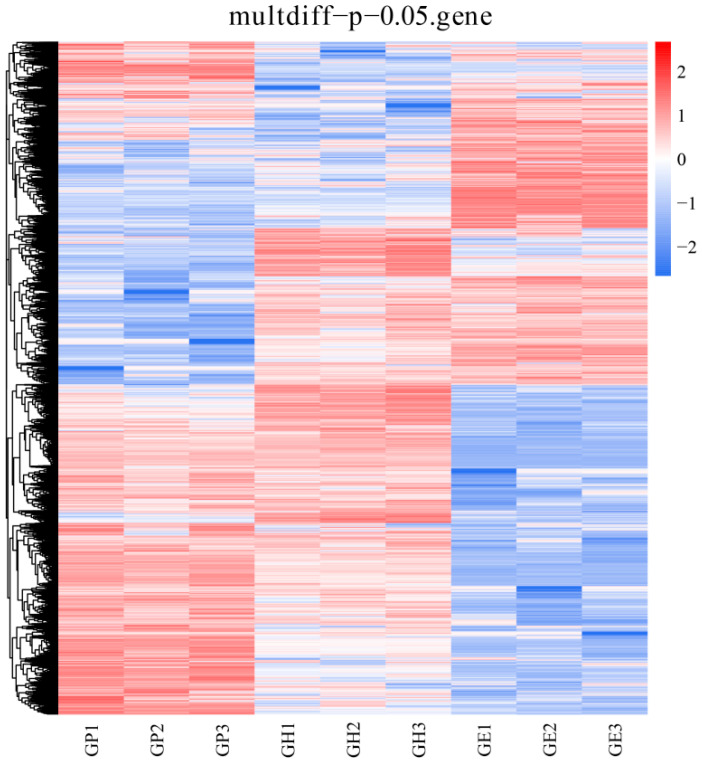
Hierarchical cluster analysis of DEGs of three groups (GP, GH and GE). Different colors represent different relative abundance of genes, where red represents higher intensity and blue represents lower intensity.

Results of hierarchical cluster analysis of DEGs showed that the gene expression pattern of the F1 hybrid generation was closer to that of the GP group, but rather different from that of the GE group (Figure 3).

### 3.1. Metabolism-Related DEG Pathway Analysis

**Figure 4:**
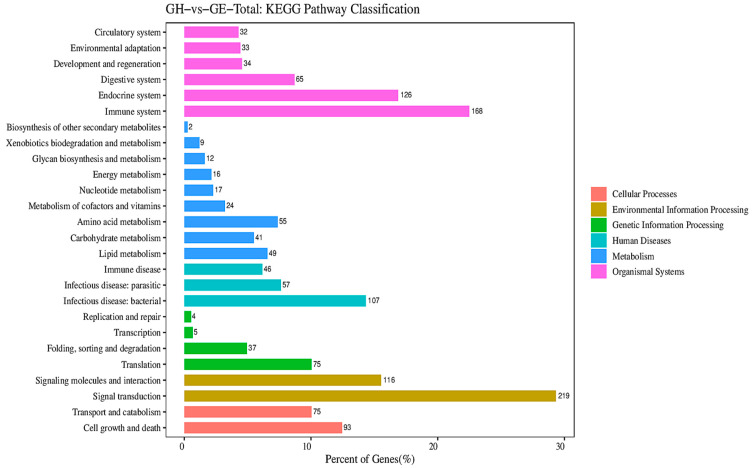
Kyoto Encyclopedia of Genes and Genomes (KEGG) pathway enrichment analyses of DEGs in muscles in the GH vs. the GE.

**Figure 5:**
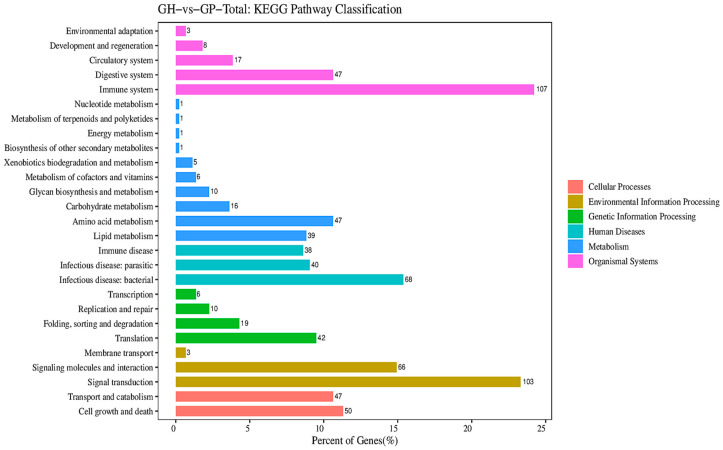
Kyoto Encyclopedia of Genes and Genomes (KEGG) pathway enrichment analyses of DEGs in muscles in the GH vs. the GP.

All DEGs were graded into five categories in KEGG pathway classification according to their biological function, such as cellular processes (GH vs. GE, 168; GH vs. GP, 97), environmental information processing (GH vs. GE, 335; GH vs. GP, 172), genetic information processing (GH vs. GE, 121; GH vs. GP, 77), human diseases (GH vs. GE, 210; GH vs. GP, 146), metabolism (GH vs. GE, 225; GH vs. GP, 127), and organismal systems (GH vs. GE, 458; GH vs. GP, 182) (Figure 4 and Figure 5). The pathways related to metabolism were mainly subdivided into eight subsets (level 2): amino acid metabolism (GH vs. GE, 55; GH vs. GP, 47), lipid metabolism (GH vs. GE, 49; GH vs. GP, 39), carbohydrate metabolism (GH vs. GE, 41; GH vs. GP, 16), metabolism of co-factors and vitamins (GH vs. GE, 24; GH vs. GP, 6), nucleotide metabolism (GH vs. GE, 17; GH vs. GP, 1), energy metabolism (GH vs. GE, 16; GH vs. GP, 1), glycan biosynthesis and metabolism (GH vs. GE, 12; GH vs. GP, 10), and xenobiotics biodegradation and metabolism (GH vs. GE, 9; GH vs. GP, 5) (Figure 4 and Figure 5).

**Figure 6:**
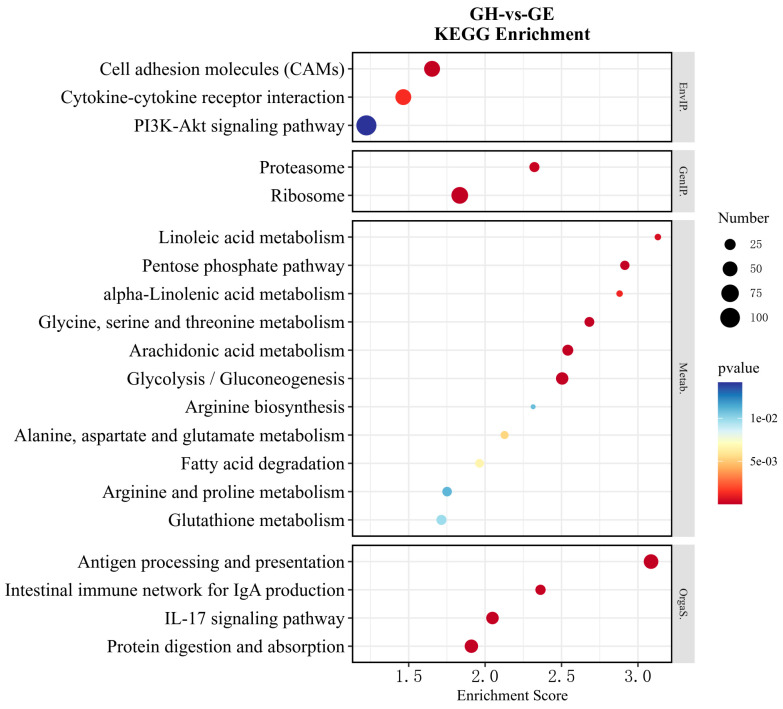
The enrichment analysis of DEGs in KEGG pathways (top 20) in the GH vs. GE. The horizontal axis in the figure is the enrichment score; the larger the bubble, the higher the number of genes enriched in the pathway; as the bubble color changes from blue to yellow to red, the enrichment *p*-value decreases and the significance increases.

**Figure 7:**
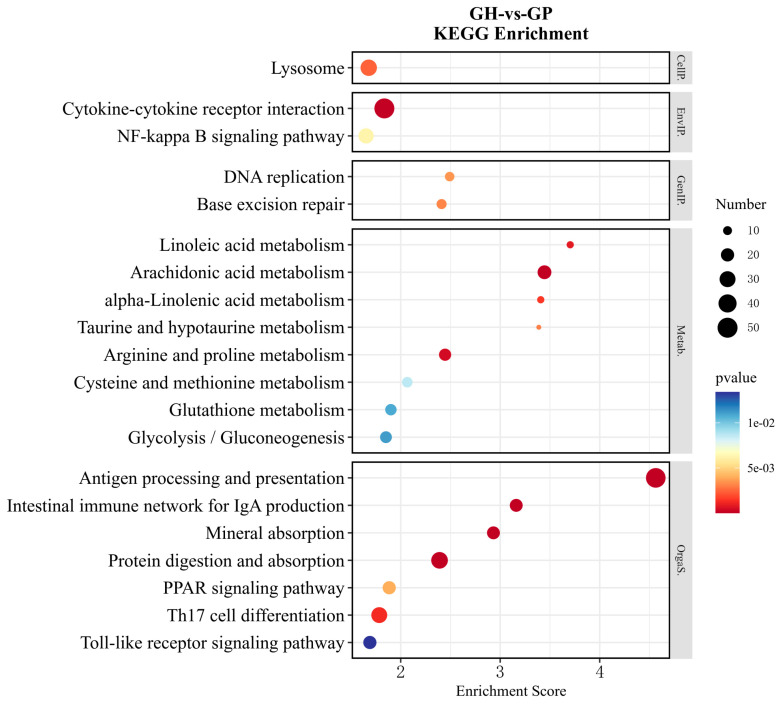
The enrichment analysis of DEGs in KEGG pathways (top 20) in the GH vs. GP. The horizontal axis in the figure is the enrichment score; the larger the bubble, the higher the number of genes enriched in the pathway; as the bubble color changes from blue to yellow to red, the enrichment *p*-value decreases and the significance increases.

Functional enrichment was also performed on the DEGs according to the above KEGG pathway classification (Figure 6 and Figure 7). Further analysis of the top 20 pathways indicated that there were 11 and 8 pathways that were directly related to metabolism in GH vs. GE and GH vs. GP, respectively. In detail, the most common metabolic pathways were glycolysis/gluconeogenesis (ko00010), arachidonic acid (ko00590), glutathione (ko00480), glycine, serine, and threonine (ko00260) metabolisms in GH vs. GE (Figure 6). The most common metabolic pathways in GH vs. GP were arachidonic acid (ko00590), arginine and proline (ko00330), glycolysis/gluconeogenesis (ko00010), and glutathione (ko00480) metabolisms (Figure 7). In addition, the PI3K-Akt signaling pathway (ko04151) was the most enriched in GH vs. GE (Figure 6), while cytokine–cytokine receptor interaction (ko04060) was the most enriched in GH vs. GP (Figure 7).

Based on the results of KEGG pathway enrichment analyses, some important metabolic pathways and growth-relative signal pathways were selected for further analysis to compare GH and GP: glycolysis/gluconeogenesis (ko00010), arginine biosynthesis (ko00220), fatty acid synthesis (ko00010), TCA cycle (ko00020), MAPK signaling pathway (ko04010), and PI3K-Akt signaling pathway (ko04151) (Table S4). The following pathways were selected for further analysis to compare GH and GE: glycolysis/gluconeogenesis (ko00010), beta-Alanine metabolism (ko00410), fatty acid genesis (ko00010), TCA cycle (ko00020), and MAPK signaling pathway (ko04010; Table S5).

### 3.2. Validation of DEGs by Quantitative Real-Time PCR (qRT-PCR)

**Figure 8:**
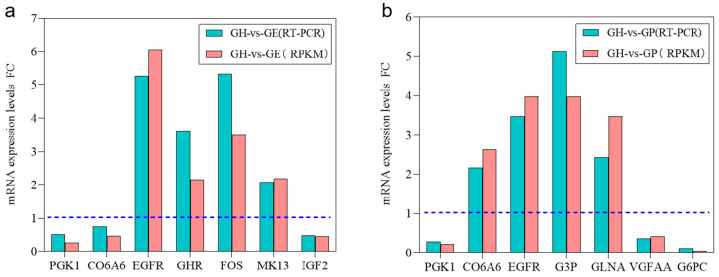
The data comparison validating DEGs determined via RT-PCR and RNA-seq. (**a**) The data comparison validating DEGs determined via RT-PCR and RNA-seq in GH vs. GE. (**b**) The data comparison validating DEGs determined via RT-PCR and RNA-seq in GH vs. GP. PGK1, proto-oncogene c-Fos; Co6a6, collagen alpha-6 (VI) chain; EGFR, epidermal growth factor receptor; GHR, growth hormone-binding protein; FOS, phosphoglycerate kinase 1;MK13, mitogen-activated protein kinase 1; IGF2, insulin-like growth factor II; G3P, glyceraldehyde 3-phosphate dehydrogenase; GLNA, glutamine synthetase QtsA; VGFAA, vascular endothelial growth factor A-A; G6PC, glucose-6-phosphatase. The pink-colored bar represents the expression levels of DEGs determined via RNA-seq in GH vs. GP and GH vs. GE, and the green-colored bar indicates the expression levels of DEGs determined via quantitative real-time PCR in GH vs. GP and GH vs. GE. Blue line indicates FC = 1 (*p* = 0.05); above the line means up-regulation (*p* < 0.05), and below the line means down-regulation (*p* < 0.05).

A subset of important DEGs in GH vs. GP (e.g., *PGK1*, *CO6A6*, *EGFR*, *CO6A6*, *G3P*, *GLNA*, *VGFAA*, *G6PC*) and GH vs. GE (e.g., *PGK1*, *CO6A6*, *EGFR*, *GHR*, *G3P*, *FOS*, *VGFAA*, *IGF2*) were randomly selected for qRT-PCR validation. The qRT-PCR primers were designed based on the mapped sequences (Supplementary Materials). The results were further compared with those generated from transcriptome sequencing. Our results showed that the data from the two different methods were consistent (Figure 8).

## 4. Discussion

Hybrid breeding is used by aquaculturists in the hopes of producing fish with target desirable traits or for the general improvement of the quality and health of fish. The overall goal of hybrid breeding is to produce offspring that perform better than both of the parental species (hybrid vigor or positive heterosis) [20]. For instance, a hybrid Hulong grouper with the physiological features of both parental species combined presents an increase in growth rate of 35.9% as compared to the maternal Brown-spotted grouper [21]. Available studies suggest that Qinghai Lake’s extreme hydrological and climatic conditions cause *G. przewalskii* to grow very slowly [16,22]. In this study, we found that the growth rate of *G. eckloni* is significantly faster than that of *G. przewalskii*. The F1 hybrid of the two species inherited the advantages of paternal *G. eckloni*, whereby its growth rate was 65% faster than that of *G. przewalskii*. Although the F1 hybrid generation had advantages over the parent *G. przewalskii*, the difference in growth rate compared to *G. eckloni* was greater by 128%. Thus, subsequent backcrossing may further improve the growth performance [23]. It is worth noting that the improved survival rate of F1 hybrid fish because of heterosis is possibly related to the enhanced adaptability of the hybrid progeny to the changing environment.

In this study, the F1 hybrid fish present heterosis in the form of fast growth. Over the years, research has demonstrated that heterosis is closely linked to molecular mechanisms [4,24,25]. For instance, the protein expression profiling of hybrid Pacific oysters (*Crassostrea gigas*) and their parents suggested that the metabolic functions of proteins play an important role in imparting a growth advantage on the hybrid progeny [24]. Here, we conducted a comparative transcriptomic study on the hybrid GH and its parents, *G. eckloni* and *G. przewalskii*. The gene expression pattern of the F1 hybrid differed less from *G. przewalskii* and more from *G. eckloni*. In addition, the results of several DEGs were also consistent with this observation. These DEGs may mainly be responsible for the difference in growth between the offspring and the parent.

### 4.1. Differences in the Metabolism of F1 Hybrid Gymnocypris and Its Parents

Previous studies have revealed the role of the glycolysis/gluconeogenesis pathway (ko00010) in promoting muscle growth, which largely involves glycolytic genes [26,27]. The activation of glycolysis/gluconeogenesis in female muscle tissues contributes to the sexual size in flatfish. Several important genes involved in glycolysis (e.g., *gpi*, *aldo*, *tpi*, *gapdh*, *bpgm*, *pgk*, *eno2*, *pk*, and *ldh*) were expressed more by several degrees in the female muscle tissues than in the male muscle tissues [27]. Furthermore, the enzyme pyruvate kinase M2 (*PKM2*) of glycolysis is an important metabolic means of mediating the effects of growth signals to promote cell proliferation; growth stimulations also lead to *PKM2* phosphorylation which regulates the conversion of protein kinase and pyruvate kinase activities [28]. In our study, the F1 hybrid fish did not present an active glycolytic gene signaling pathway, but it clearly had better growth compared to *G. przewalskii*. So, there is reason to doubt that the regulation of fish growth may directly affect other growth-related pathways, such as the growth hormone insulin-like growth factor (*IGF*) system, which is a key promoter of growth in vertebrates [4].

Oxidative phosphorylation (ko00190) is the main pathway of energy production in living organisms under aerobic conditions; thus, it is critical to maintaining the activities of life, growth, and development in fish. It has been reported that impaired oxidative phosphorylation in hepatic mitochondria can lead to growth retardation in rats [29]. Our results show that the DEGs between the F1 hybrid and *G. przewalskii* were not enriched in the oxidative phosphorylation pathway, while four DEGs related to oxidative phosphorylation were up-regulated in the F1 hybrid fish compared to *G. eckloni*. These genes were mainly divided into two categories: one category was related to proton reflux ATP synthase genes and the other category included two DEGs related to the oxidative respiratory chain of electrons; these DEGs were closely involved in the generation of energy and their up-regulation may indicate that the F1 hybrid fish were more vigorous in energy metabolism and had greater energy demand for growth than their parents.

Similarly, three DEGs were enriched in the citrate cycle (TCA) that is responsible for producing ATP (ko00020); this differential expression was observed only between the F1 hybrid and *G. eckloni* for *IDH* (isocitrate dehydrogenase) and *IDH2*, which were highly expressed; isocitrate dehydrogenase is a key rate-limiting enzyme in the TCA cycle. Moreover, previous reports have shown that IDH expression has a positive correlation with growth [30]. We observed that the DEGs related to energy metabolism in F1 hybrid fish were expressed more, suggesting that their energy supply or consumption was stronger than that of *G. eckloni*.

Arginine can enhance immunity and deepen nutrition [31]. In F1 hybrid generation, DEGs involved in the arginine signal pathway (ko00220) were highly expressed as compared to *G. eckloni* and *G. przewalskii*. This indicated the benefit of hybridization. The arachidonic acid pathway plays a key role in cardiovascular biology, carcinogenesis, and many inflammatory diseases [32]. In our study, the polyunsaturated fatty acid 5-lipoxygenase (*LOG5*) was highly expressed only in the F1 hybrid. *LOG5* converts arachidonic acid into several inflammatory mediators in the presence of a 5-lipoxygenase-activating protein [33]. Some studies have shown that the overexpression of *LOG5* in tumor tissue leads to breast cancer [34], while its function has not been reported in fish.

### 4.2. Differences in the Relative Growth Pathways of F1 Hybrid Gymnocypris and Its Parents

In the modulation of skeletal muscle growth in teleost fish, MAPK, PI3K-Akt, and mTOR are important signaling pathways involved in fish growth [4,35]. In a study on zebrafish, the MAPK signaling pathway (ko04010) was strongly activated through the expression of FGF, which promoted the up-regulation of Ras and MKP, enhanced phosphorylation, and led to an increase in cell proliferation [36]. In many respects, the FGFR appears to be similar to other growth factor receptors [37]. However, compared with *G. przewalskii*, the DEG of *fgfr* was less expressed in the F1 hybrid fish; thus, the function of *fgfr* in *Gymnocypris* fish species requires further investigation. Interestingly, the DEG *IGF2* in the MAPK pathway was found in the F1 hybrid fish and *G. eckloni*, where it was highly expressed in *G. eckloni*. Previous studies have shown that signaling molecules may be activated by IGFs and they are involved in fish muscle growth [12]. IGFs stimulate muscle growth in fish by promoting the proliferation of myogenic cells, protein synthesis, and hypertrophy; particularly, *IGF1* and *IGF2* proteins activate MAPK/ERK and PI3K/AKT signaling pathways in fish skeletal muscles [35]. *IGF2* has a dramatic and direct effect on muscle growth in fish. For instance, it activates these pathways in the myogenic cells of the gilt-head sea bream [38] and rainbow trout [39]. Some studies have suggested that IGF2 is more potent than IGF1 in stimulating muscle growth in fish species [38,40]. *IGF2* may also contribute to the growth superiority of the hybrid grouper by enhancing protein and glycogen synthesis [4]. The down-regulation of DEGs involved in the glycolysis/gluconeogenesis pathway might be controlled via the down-regulation of *IGF2*. Thus, the difference in the growth of *G. eckloni* and the F1 hybrid fish may be influenced by the differential expression of *IGF2*. Uncovering the various growth-related genes and pathways involving *IGF2* could serve as an essential baseline for genetic improvement and selection of superior *G. przewalskii* and other *Gymnocypris* species for hybridization.

In addition, growing evidence suggests that the PI3K-Akt pathway (ko04151) may play an important role in eliciting its effects via MAPK signaling [41]. Collagen VI is a major extracellular matrix (ECM) protein that helps maintain the functional integrity of skeletal muscles [42]. The Col6a1 gene has been reported to be involved in ECM remodeling during muscle fibrosis, and Col6a1/mice and collagen VI-deficient zebrafish display a myopathic phenotype [42]. Moreover, genes in the growth arrest and DNA damage (*GADD*)-inducible family are often up-regulated in response to various environmental stresses and drug therapies. GADD45α was the first stress-inducible gene that was determined to be up-regulated by p53. When *GADD45α* is deleted or repressed, cells show uncontrolled proliferation [43]. In our study, the down-regulation of *GADD45α* in F1 hybrid fish could have caused the acceleration of cell proliferation, and it may be the reason why the F1 hybrid fish grow faster than *G. przewalskii*. The mTOR signaling pathway (ko04150) is important in cell growth and development, as well as in protein synthesis [44]. In this pathway, some DEGs related to cell growth were observed between the F1 hybrid and *G. eckloni*, such as S6 kinases (*S6Ks*) [12,45] and phosphatidylinositol 3,4,5 [46]. Compared to *G. przewalskii*, these two DEGs were highly expressed in the F1 hybrid fish and are speculated to be involved in the regulation of the rapid growth of fish [12].

## 5. Conclusions

In summary, growth performance analyses showed that *G. eckloni* grew faster than *G. przewalskii*, and the F1 hybrid fish presented an even more significant growth advantage compared to *G. przewalskii*. Based on analysis of RNA-seq data, our results revealed that gene transcription has a significant impact on F1 hybrid *Gymnocypris* and its parents. In addition, the genes in the F1 hybrid group showed fewer differences with *G. przewalskii* but more differences with *G. eckloni*. KEGG enrichment results indicated that most DEGs were related to a variety of molecular functions, such as glycolysis/gluconeogenesis, arachidonic acid metabolism, citrate cycle, MAPK, PI3K-Akt, and mTOR signal pathways. Further analysis revealed that the difference in the growth of *G. eckloni* and the F1 hybrid fish may have an important relationship with the differential expression of *IGF2*. Growth-related genes and pathways were uncovered that involved IGF2; these can serve as an essential baseline for the genetic improvement and selection of *G. przewalskii* and other *Gymnocypris* species.

## Figures and Tables

**Table 1:** The growth trait measurements of F1 hybrid *Gymnocypris* and its parents.

		GH	GE	GP
Length	initial	1.52 ± 0.08	1.66 ± 0.12	1.49 ± 0.05
	terminal	8.49 ± 1.21 ^b^	11.73 ± 1.00 ^a^	7.23 ± 0.40 ^c^
Weight	initial	1.78 ± 0.13	1.82 ± 0.19	1.64 ± 0.07
	terminal	9.41 ± 2.18 ^b^	20.69 ± 5.12 ^a^	5.54 ± 0.71 ^c^
Weight rate (%)		428.65 ± 52.28 ^b^	977.60 ± 87.62 ^a^	259.74 ± 31.45 ^c^
Survival rate (%)		80.50 ± 1.00 ^a^	72.33 ± 1.53 ^b^	68.17 ± 2.93 ^c^

Note: GH is the F1 hybrid of two *Gymnocypris*, GP is the *G. przewalskii*, and GE is the *G. eckloni.* Values (expressed as mean ± SD, n = 15) with different letters (a, b, c) within the same line are significantly different (*p* < 0.05).

## Data Availability

The data presented in this study are available on request from the corresponding author. The data are not publicly available due to privacy.

## Supplementary Materials

The following supporting information can be downloaded at: https://www.mdpi.com/article/10.3390/genes15020182/s1.
