# Supplementary material for: Transcriptome Analysis Reveals the Molecular Mechanisms Underlying Growth Superiority in a Novel Gymnocypris Hybrid, Gymnocypris przewalskii ♀ × Gymnocypris eckloni ♂
Source: Genes (Basel). 2024 Jan 29;15(2):182. doi: 10.3390/genes15020182 (PMC10888472; doi:10.3390/genes15020182)
Supplement: Supplementary file 1 [file genes-15-00182-s001.zip › genes-2793364-supplementary.pdf]

Table S1 The proximate composition of expanded formula feed

Nutrient compositions	Content
Crude protein	≥38.0%
Crude fat	≥6.0%
Crude fiber	≤4.0%
Crude ash	≤17.0%
Moisture	≤11.0%
Phosphorus	≥1.2%
Lysine	≥1.8%

Table S2. The primer for RT-PCR of validation DEGs.

Gene	Primer (5'-3')
PGK1 F	TTGGACAAGCTGGACGTGAAGG
PGK1 R	GTGGCTCATAAGGACAACGGACTT
VGFAA F	TGCGGCTCTCCTTCATCTGTCT
VGFAA R	AGGTGTGCTCGATCTCGTCAGG
GHR F	CGCCCACCACAATAACCCAAAC
GHR R	TCCAGTCCACGCCATCTACCAT
IGF2 F	GTGCTCACACGCTCTTCCAGTT
IGF2 R	CGTTCGGCGGCTTCTTTGTTCT
EGFR F	GCACAGCCAGACGATCACTACA
EGFR R	AGCACATAGCCACCGACTTCTT
CO6A6 F	ACGCTTCGCTTACGCCTTCAT
CO6A6 R	GCTCTTCGGTGGGTGCTTCA
G6PC F	TCAACAGCTCAACGCCGCATA
G6PC R	CGAGGATCGAGGTGACCAATGT
GLNA F	CGGTTCATCCTGCGGACAAG
GLNA R	GCGGGTGTTACTTTATCGGCTA
MK13 F	CCTGGCTGTGAACCAAGACTGT
MK13 R	CTGTCTGCGTGTAGTGCATCCA
EF1α F	GTATTACCATTGACATTGC
EF1α R	CTGAGAAGTACCAGTGAT

Table S3. Sequencing statistics of RNA-Seq library from F1 hybrid *Gymnocypris* and its parents.

Sample	RawReads (M)	RawBases (G)	CleanReads (M)	CleanBases (G)	ValidBases (%)	Q30 (%)	GC (%)
GE1	49.49	7.42	49.12	7.08	95.35	93.4	49.12
GE2	48.89	7.33	48.53	7.03	95.83	93.78	49.13
GE3	47.35	7.1	47.01	6.75	95.02	93.88	49.04
GH1	50.28	7.54	49.94	7.17	95.06	93.79	48.96
GH2	51.37	7.71	51.02	7.39	95.87	93.53	48.85
GH3	48.65	7.30	48.35	7.04	96.41	94.35	48.97

GP1	48.94	7.34	48.60	6.95	94.67	93.86	48.57
GP2	48.75	7.31	48.40	7.00	95.78	93.90	48.77
GP3	49.18	7.38	48.86	7.08	95.93	93.70	48.74

Table S4. The metabolic and environmental information processing related differentially expressed genes (DEGs) identified in muscles of GH vs GP by transcriptome.

Swissprot annotation	Swissprot id	logFC	p-value
**Metabolism**			
**Glycolysis / Gluconeogenesis(ko00010)**			
Alpha-enolase Eno-1	P17182	-2.3926	0.0003
Fructose-1,6-bisphosphatase 1 FBP1	Q3SZB7	-2.2772	0.0008
Glucose-6-phosphatase G6PC	Q19KA1	-6.5752	4.58E-08
L-lactate dehydrogenase A chain Ldh-1	P06151	-1.9633	0.0055
Phosphoenolpyruvate carboxykinase	P05153	-5.2828	0.0057
Phosphoglycerate kinase 1 Pgk-1	P09411	-2.1472	0.0001
Aalcohol dehydrogenase (NADP+)	Q6AZW2	1.4892	1.77E-10
Glyceraldehyde-3-phosphate dehydrogenase GAPI	Q94469	1.9903	1.17E-06
Triosephosphate isomerase	Q9GTX8	1.8906	6.26E-05
**Arginine biosynthesis（ko00220）**			
Alanine aminotransferase 2-like im:6791811	Q6NYL5	-4.9577	0.0018
Arginase-1 ARG1	P05089	-4.2122	3.59793E-05
Arginase, non-hepatic 2 arg2	Q91554	-1.1995	2.65507E-60
Glutamine synthetase QtsA-14381	Q4R7U3	1.7976	0.0004
**Cysteine and methionine metabolism(ko00270)**			
Putative adenosylhomocysteinase 3 Ahcyl2	Q68FL4	-7.0535	1.07E-06
S-adenosylhomocysteine hydrolase-like protein 1	O43865	-5.7384	5.16E-06
Adenosylhomocysteinase PC000295.02.0	Q4XZZ5	4.2795	9.77E-05
**Lysine degradation（ko00310）**			
Histone-lysine N-methyltransferase SMYD1	Q8NB12	2.4899	1.3209E-61
Histone-lysine N-methyltransferase SUV39H1 KMT1A	O43463	1.0269	0.0007
N-lysine methyltransferase KMT5A-A zgc:153719	Q071E0	1.1683	0.0029
**Glutathione metabolism（ko00480）**			
Glutathione peroxidase 3	P23764	-2.4685	2.0150E-59
Glutathione-specific gamma-glutamylcyclotransferase 1	Q5SPB6	-1.1686	5.21E-112
Glutathione S-transferase theta-1	P20135	-5.1711	4.0882E-07
**Arachidonic acid metabolism(ko00590)**			
BCL2/adenovirus E1B 19 kDa protein-interacting protein 3	Q12983	-1.0419	1.33E-77
Prostaglandin E synthase PTGES	Q95L14	-1.1358	1.20E-27
Serine/threonine-protein kinase Sgk1 SGK	Q6U1I9	-1.0708	1.85E-14

Polyunsaturated fatty acid 5-lipoxygenase LOG5	P09917	3.6118	3.03E-113
**Environmental Information Processing**			
**MAPK signaling pathway（ko04010）**			
Fibroblast growth factor receptor 4 fgfr4	Q90413	-4.6288	0.001
Growth arrest and DNA damage-inducible protein GADD45	Q2KIX1	-1.7252	8.11E-53
Mitogen-activated protein kinase kinase kinase3	Q8IVH8	-6.1067	0.0062
Heat shock protein beta-1 HSPB1	Q00649	2.2259	2.61E-249
Receptor-type tyrosine-protein kinase FLT3 Flk-2	Q00342	2.1681	0.0003
**mTOR（ko04150）**			
4F2 cell-surface antigen heavy chain	P10852	-1.5590	4.20E-205
E3 ubiquitin-protein ligase rnf152	Q58EC8	1.4173	0.0041
Phosphatidylinositol 3,4,5	P60484	2.3678	4.95E-37
Ribosomal protein S6 kinase RPS6	P51812	1.0122	0.0053
**PI3K-Akt signaling pathway(ko04151)**			
Induced myeloid leukemia cell differentiation protein Mcl-1	Q8HYS5	-1.0992	1.50E-116
Osteopontin Eta-1	P10923	-3.3597	1.99E-14
Collagen alpha-6(VI) chain Col6a6	Q8C6K9	1.3966	0.0052
Laminin subunit beta-4 lamb4	Q8JHV6	1.6326	0.0050

Table S5. The metabolic and environmental information processing related differentially expressed genes (DEGs) identified in muscles of GH vs GE by transcriptome.

Swissprot annotation	Swissprot id	log2FC	p-value
**Metabolism**			
**Glycolysis / Gluconeogenesis(ko00010)**			
Fructose-1,6-bisphosphatase isozyme 2 FBP2	Q9N0J6	-1.4285	4.74E-106
Phosphoglucomutase-1 PGM1	Q08DP0	-2.5161	3.37E-07
Phosphoglycerate kinase 1 Pgk-1	P09411	-1.9136	0.0039
Pyruvate kinase PKM	Q9CWB1	-1.6209	0.0064
Glyceraldehyde-3-phosphate dehydrogenase 2	Q5MJ86	1.3699	2.30E-92
**Oxidative phosphorylation(ko00190)**			
ATP synthase subunit beta, mitochondrial .	Q25117	7.7275	3.15E-08
Cytochrome c oxidase subunit 6B1 COX6B	P00429	1.6992	4.04E-13
V-type proton ATPase 16 kDa proteolipid subunit ATP6C	P27449	1.3183	1.96E-19
V-type proton ATPase catalytic subunit A Atp6a1	P50516	6.7461	2.10E-06
**Citrate cycle (TCA cycle)(ko00020)**			
Malate dehydrogenase, cytoplasmic RCJMB04_2g5	Q5ZME2	-1.0196	7.31E-66
Isocitrate dehydrogenase [NADP], mitochondrial IDH	Q4R502	7.1973	8.31E-06
Isocitrate dehydrogenase [NADP], mitochondrial IDH2	P33198	7.1402	1.32E-06
**Arginine biosynthesis(ko00220)**			
Arginase-2, mitochondrial ARG2	P78540	3.8616	0.0027
Argininosuccinate synthase zgc:92051	Q66I24	1.9247	7.63E-38
Glutamine synthetase QtsA-14381	Q4R7U3	6.6598	2.08E-08

**Cysteine and methionine metabolism（ko00270）**			
Adenosylhomocysteinase PC000295.02.0	Q4XZZ5	7.0442	4.21E-07
S-adenosylmethionine synthase isoform type-1	Q00266	4.8893	0.0001
**Lysine degradation(ko00310)**			
Histone-lysine N-methyltransferase ASH1L	Q9NR48	-1.0952	0.0032
Histone-lysine N-methyltransferase SMYD1	Q8NB12	1.5121	1.63E-90
Gamma-butyrobetaine dioxygenase Bbh	Q9QZU7	2.1703	1.06E-06
**Arachidonic acid metabolism(ko00590)**			
Polyunsaturated fatty acid 5-lipoxygenase LOG5	P09917	8.9552	1.91E-63
Prostacyclin synthase ptgis	F1RE08	1.4724	5.21E-15
Prostaglandin G/H synthase 2 COX2	P70682	1.5627	2.38E-71
**Environmental Information Processing**			
**MAPK signaling pathway（ko04010）**			
Guanine nucleotide-binding protein	Q9UBI6	-2.5348	1.22E-10
Insulin-like growth factor II IGF2	Q02816	-1.1293	1.54E-08
Mitogen-activated protein kinase kinase kinase 3	Q8IVH8	-6.8918	4.65E-05
Dual specificity protein phosphatase 1-A	Q91790	1.3833	1.68E-145
Ephrin type-A receptor 2 EPHA2	Q1KL86	1.8650	3.60E-25
